# Supplementary material for: STIM1 transmembrane helix dimerization captured by AI-guided transition path sampling
Source: Proc Natl Acad Sci U S A. 2025 Aug 26;122(35):e2506516122. doi: 10.1073/pnas.2506516122 (PMC12415195; doi:10.1073/pnas.2506516122)
Supplement: Supplementary file 1 — Appendix 01 (PDF) [file pnas.2506516122.sapp.pdf]

Supporting Information for

STIM1 transmembrane helix dimerization captured by AI-guided transition path sampling

Ferdinand Horvath, Hendrik Jung, Herwig Grabmayr, Marc Fahrner, Christoph Romanin, Gerhard Hummer

Corresponding Author name.
E-mail: gerhard.hummer@biophys.mpg.de

This PDF file includes:

Figs. S1 to S15
Legends for Movies S1 to S3

Other supporting materials for this manuscript include the following:

Movies S1 to S3

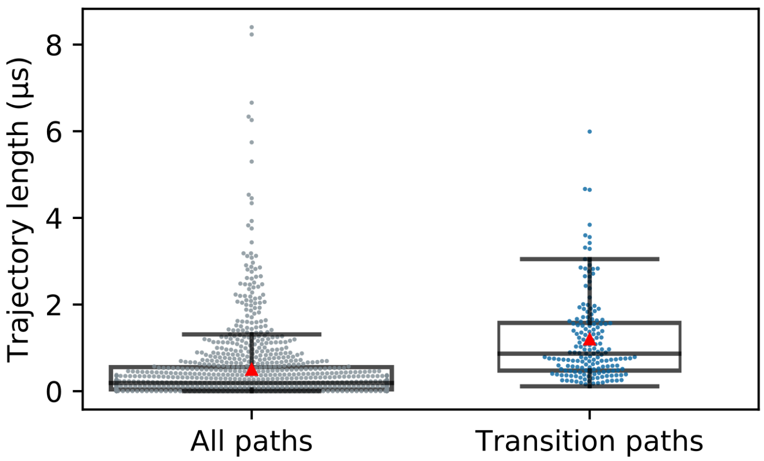

Fig. S1. Lengths of trajectories created using transition path sampling. For optimally chosen initial conformations, the probability of sampling a transition path is 0.5. Therefore, the sampled transition paths are a subset of all produced paths. Mean trajectory durations are indicated by a red triangle (1.19 μs in the case of all transition path durations).

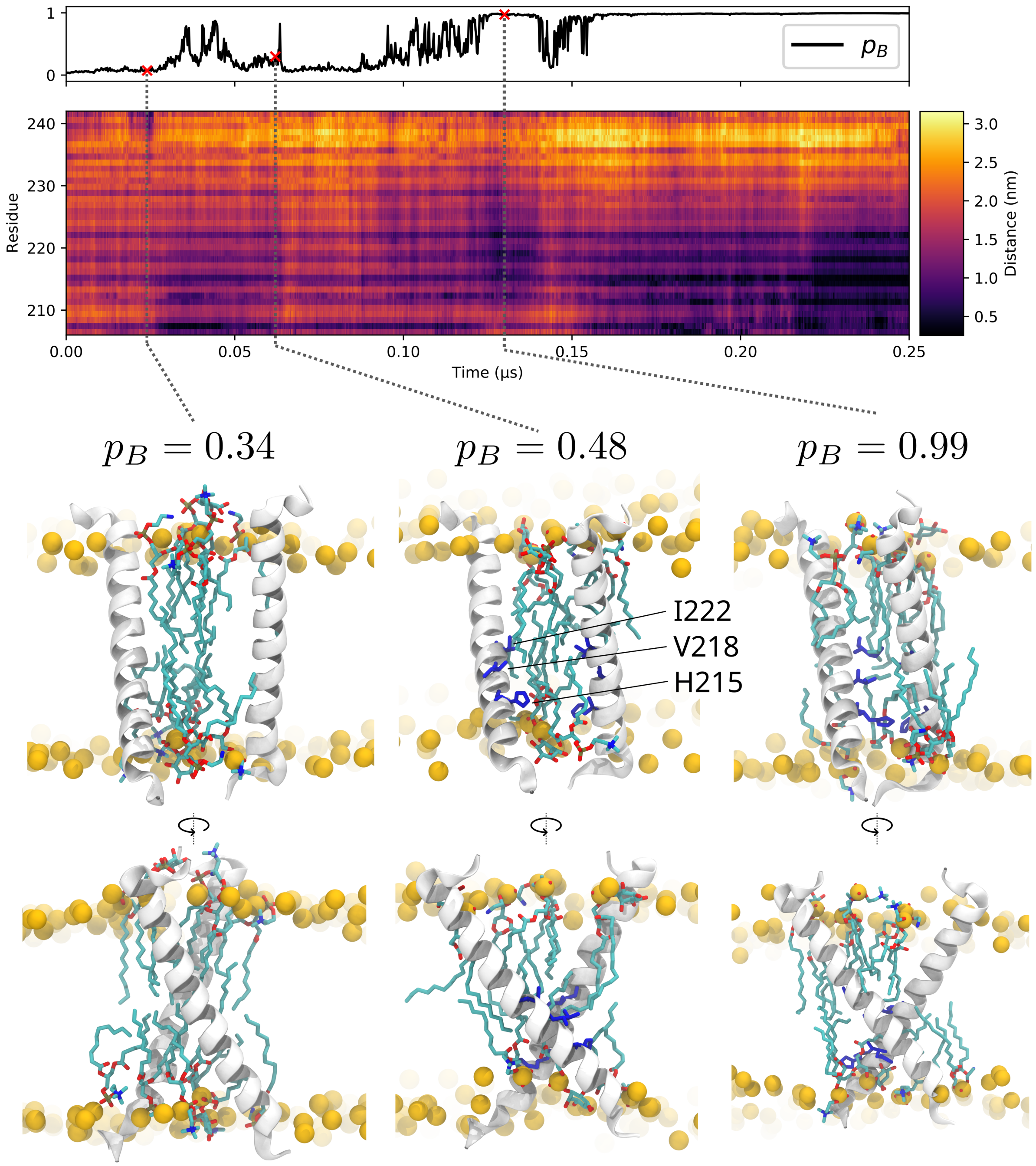

Fig. S2. Exemplary transition path. Top: $p_B(t)$ time series. Middle: Distance trajectory map. For each residue in monomer A, the distance is calculated as the shortest interhelical distance to any residue in monomer B. Bottom: Three snapshots illustrating the formation of a X_{BSE} bound state with front and side views. Residues forming interhelical contacts in the final state are highlighted in blue. Lipids in between the two helices are shown in licorice representation.

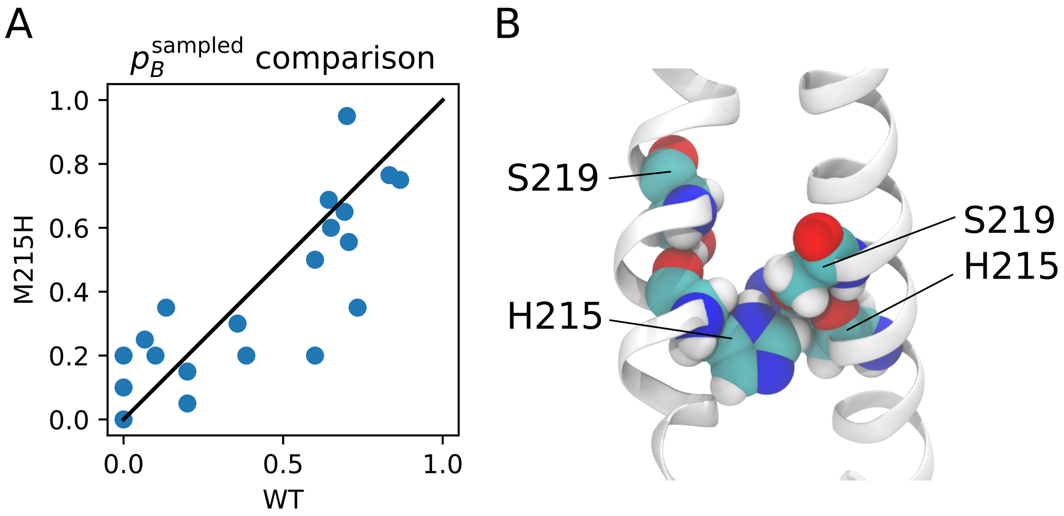

Fig. S3. Mutant M215H versus WT. (A) Sampled frequency of dimerization p_B^{sampled} for the STIM1-TM WT and the M215H mutant. Each data point represents a pair of starting conformations that were prepared identically except at position 215, where the sidechain contained either histidine (y axis) or methionine (x axis). p_B^{sampled} is the frequency with which simulations launched from these initial conformations reached the dimerized state before reaching the separated state. (B) The mutated position H215 fits well into the groove created by the SxxxG binding motif, resulting in strong interhelical van-der-Waals interactions.

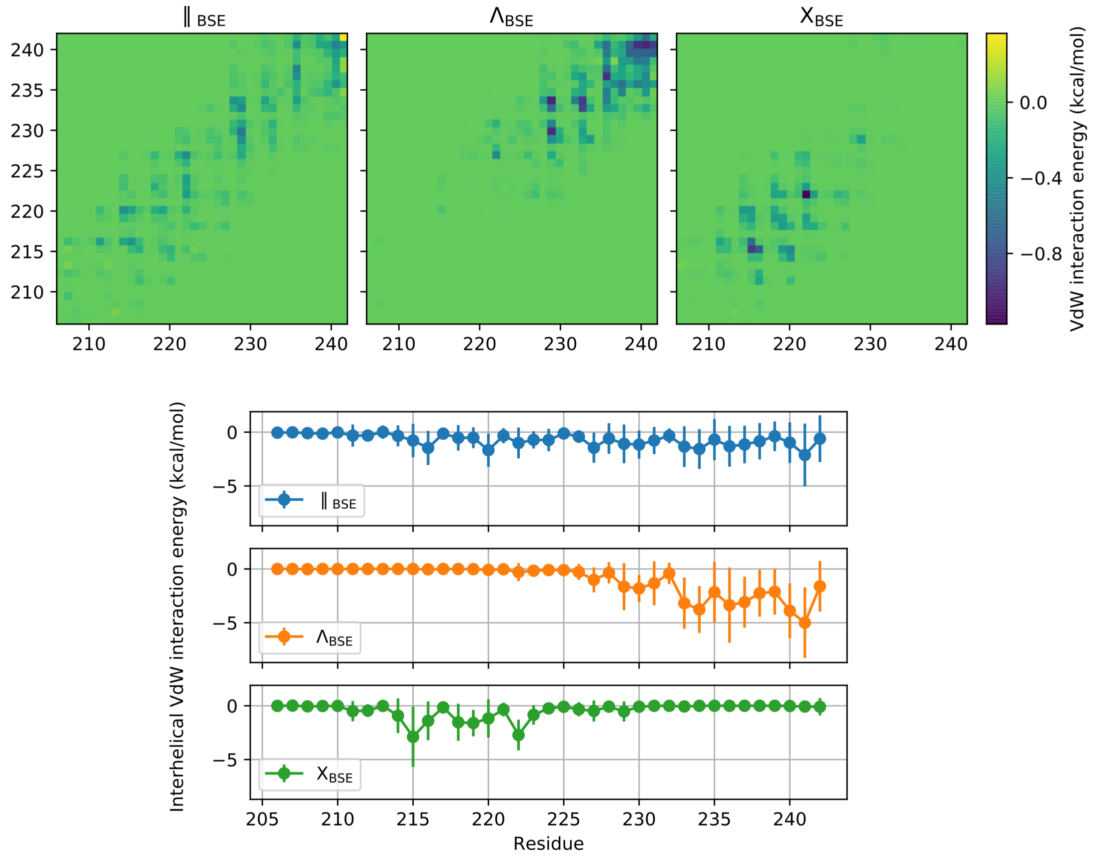

Fig. S4. Van-der-Waals interactions in the bound state. Top: Mean interhelical van-der-Waals interaction energy calculated for the three BSE clusters. Bottom: Total interhelical van-der-Waals interaction energy for the three BSE clusters.

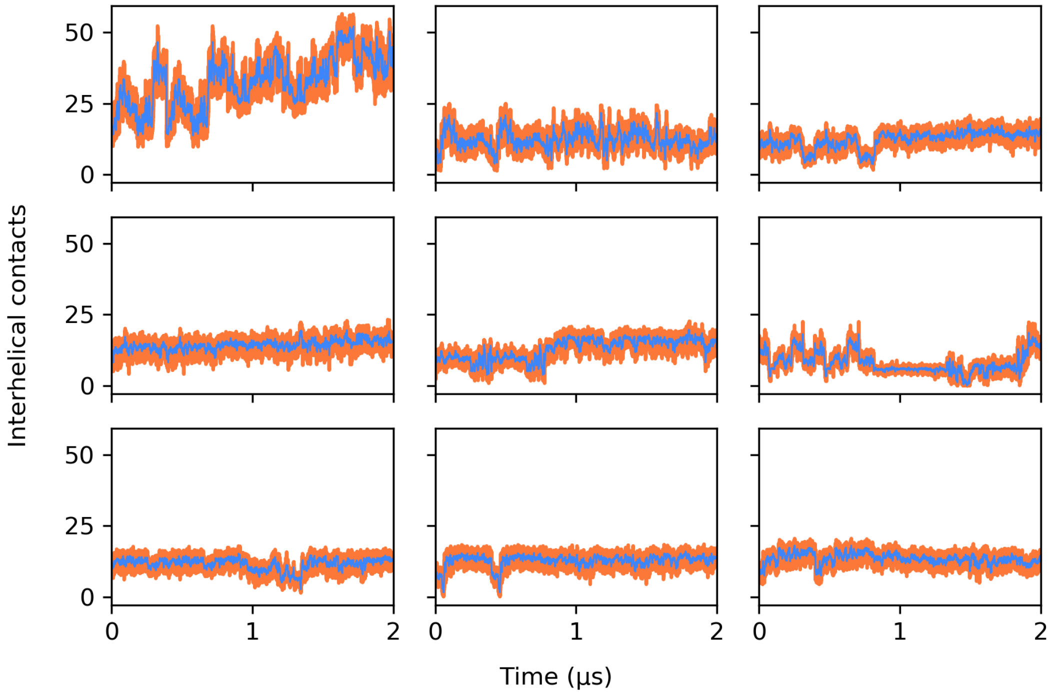

Fig. S5. Number of contacts for 9 extended trajectories testing the stability of the obtained dimerized configurations. Blue traces indicate the moving average with a time window of 2 ns. While some rearrangement after dimerization is expected, our aim is to verify that structures which are classified as dimerized by our state function do not readily dissociate when simulations are extended.

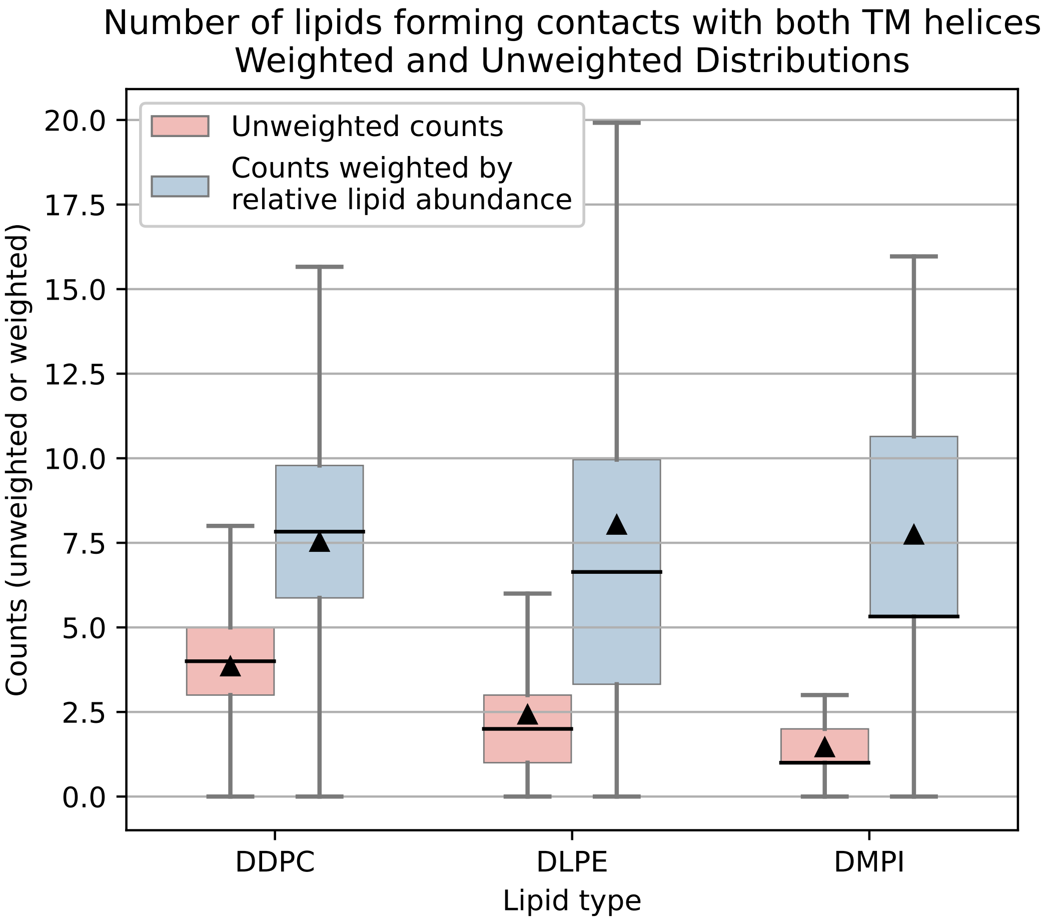

Fig. S6. Lipids associating with dimerized structures analyzed by lipid type. To check for preferential lipid aggregation around the dimer, we calculated the number of lipids that form contacts with both TM helices using a cutoff distance of 0.5 nm across all structures in the bound state ensemble. Red boxes represent unweighted lipid counts, blue boxes represent lipid counts corrected for the relative abundance of the three lipid types (box: interquartile range; line: median; triangles: mean; whiskers: range).

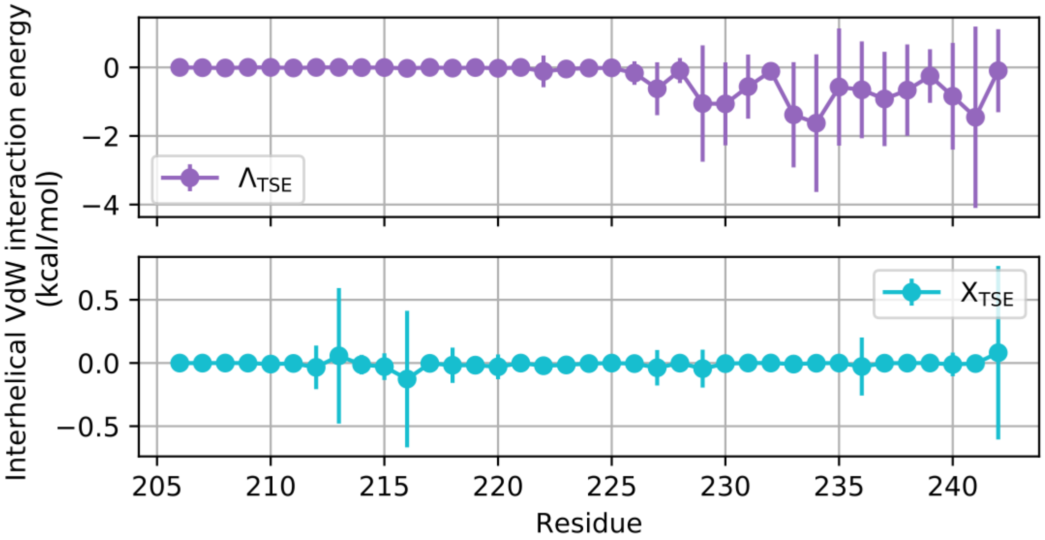

Fig. S7. Total interhelical van-der-Waals interaction energy for the two TSE clusters.

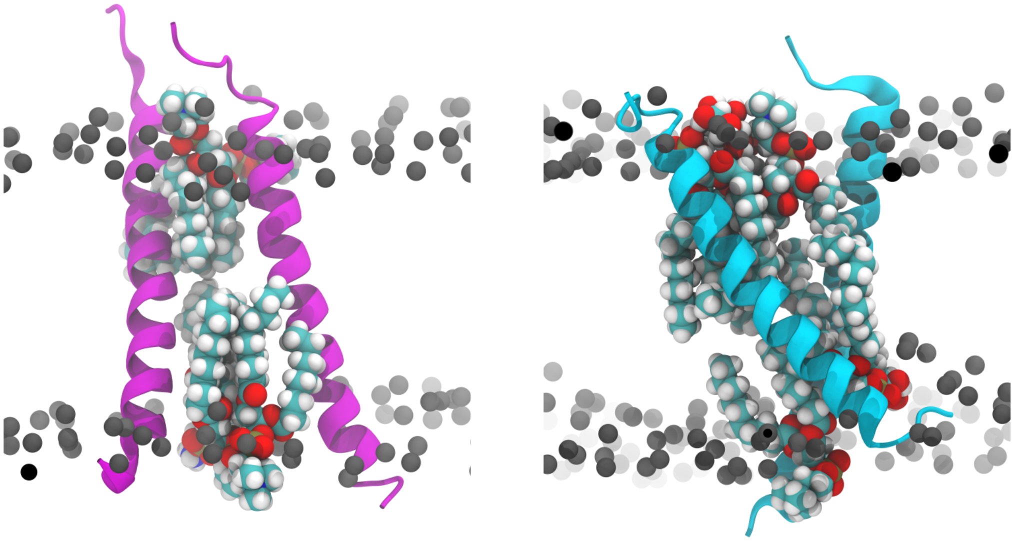

Fig. S8. Two exemplary transition state structures from the Λ^{TSE} (left) and X^{TSE} (right) clusters with lipids separating the two TM helices highlighted in spacefilling representation.

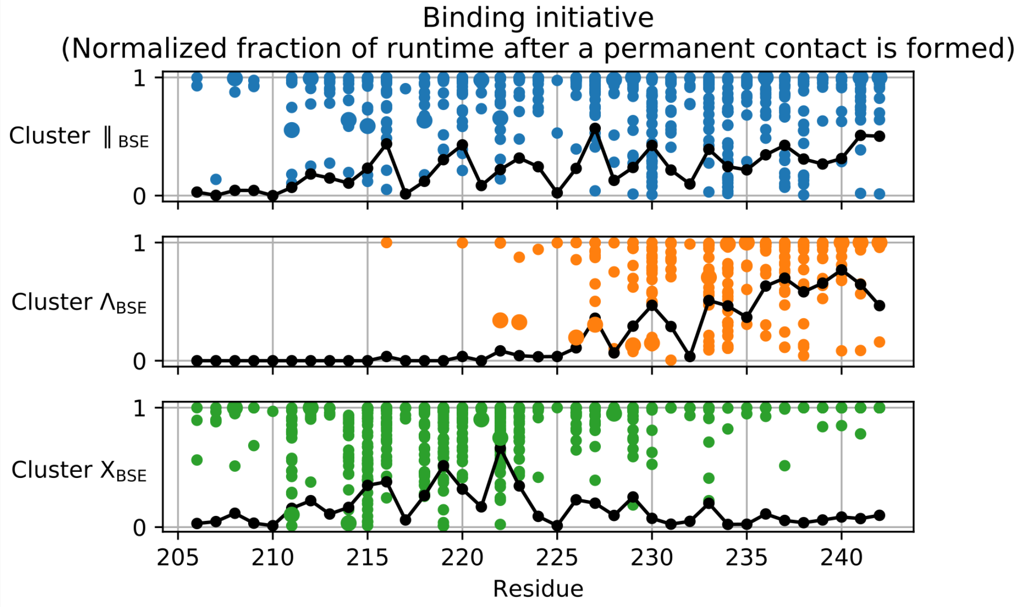

Fig. S9. Binding initiative for the TPs feeding into the three BSE clusters. Binding initiative is defined as the normalized fraction of TP runtime that remains after a residue forms a permanent interhelical contact. A value of 1 indicates that a residue is the first one to engage in an interhelical contact, a value of 0 indicates that it is last. Black lines indicate the mean taken over all TPs corresponding to the respective BSE cluster. Note that transitions feeding into the three respective dimerized states clearly differ with regards to the residues that first establish inter-monomeric contacts. This indicates that the three dimerized states X_{BSE}, Λ_{BSE} and $\|_{\mathrm{BSE}}$ do not result from a rearrangement at the very end of the transition, but that they indeed result from distinct dimerization pathways.

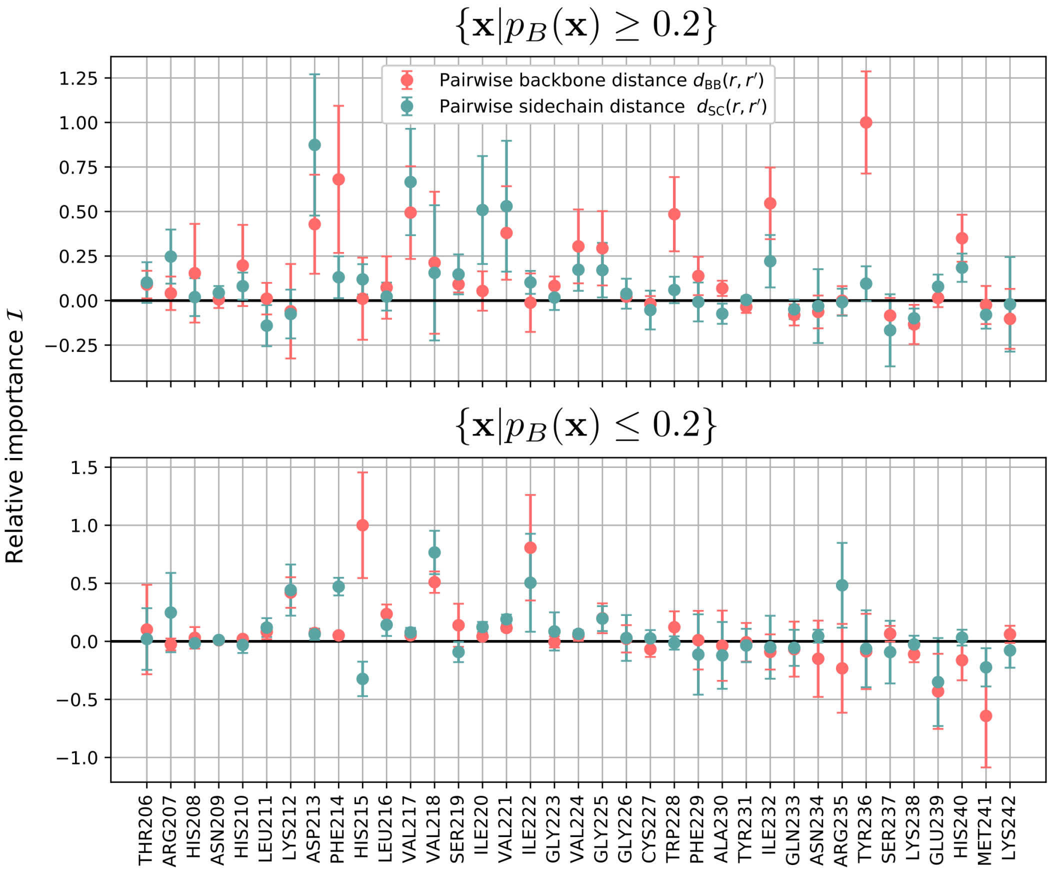

Fig. S10. Comparison of input importance analysis scores calculated for the datasets comprising shooting points $\mathbf{x}$ with $\{\mathbf{x}|p_B(\mathbf{x}) \geq 0.2\}$ and $\{\mathbf{x}|p_B(\mathbf{x}) \leq 0.2\}$, respectively. Error bars indicate the standard deviation of the normalized loss difference with respect to the reference loss obtained in 100 random permutations of the respective distance descriptor. The top panel corresponds to Figure 3A.

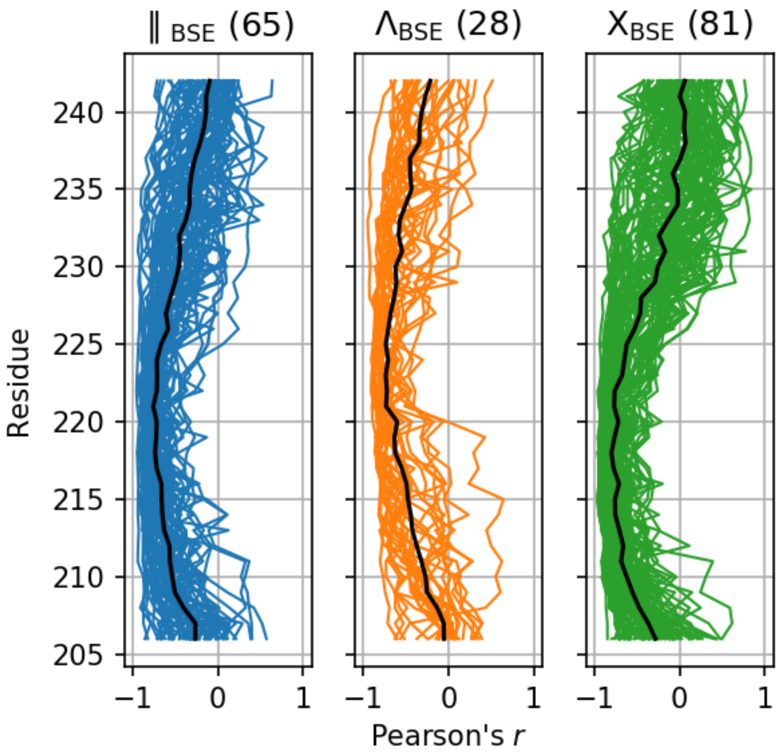

Fig. S11. Pearson's r correlation between $p_B(t)$ and the distances $d_{\mathrm{SC}}(r, r'; t)$ between equivalent residues r and r' in the two helices. Black lines indicate the average correlation for each cluster. Bracketed numbers indicate the size of the respective BSE clusters.

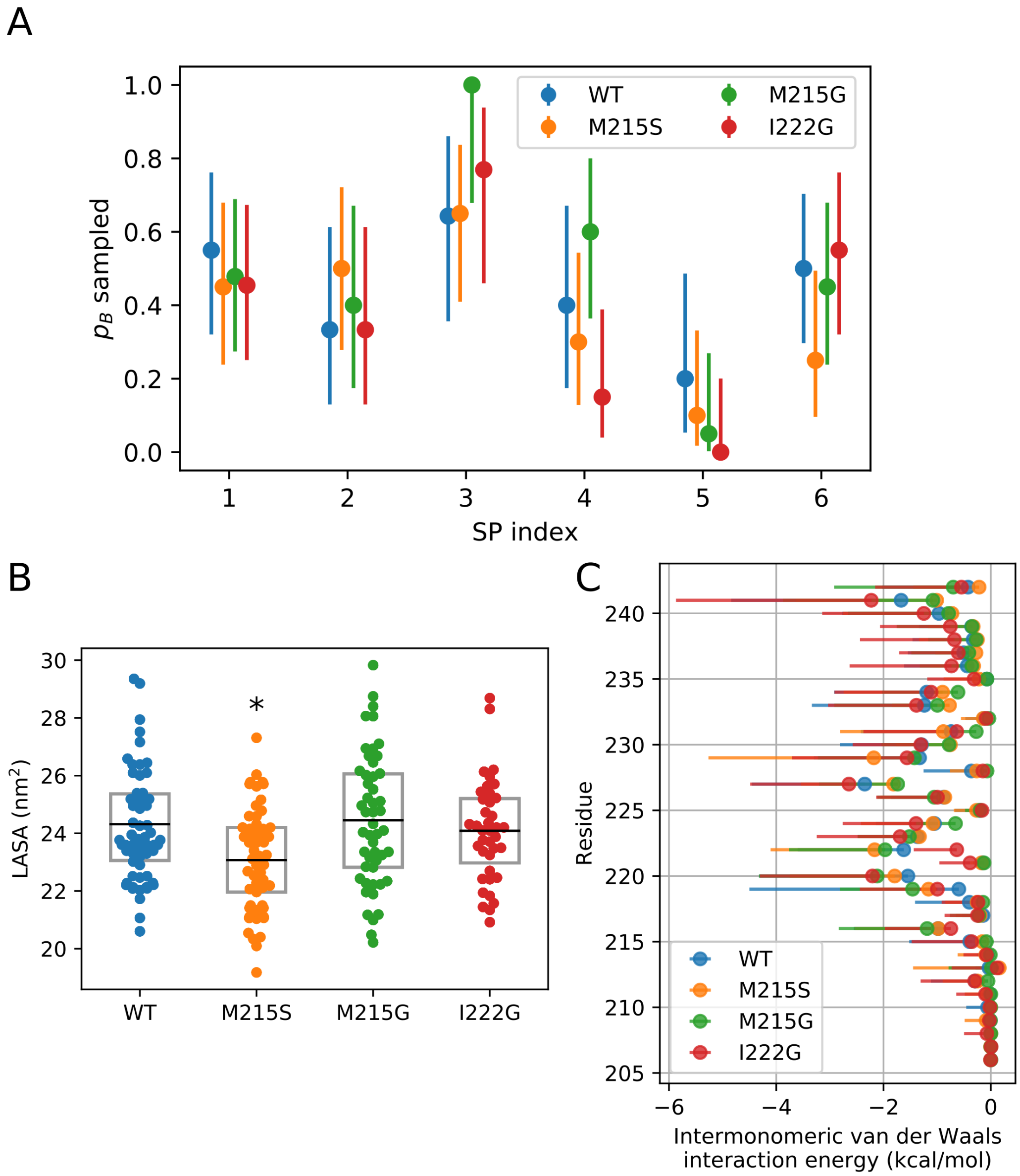

Fig. S12. Mutants M215G, M215S and I222G. (A) Sampled probability of dimerization $p_B(\mathbf{x})$ for the STIM1 WT and STIM1 mutants M215G, M215S and I222G for six distinct shooting points (SPs). Error bars indicate the 95% binomial proportion confidence interval. (B) Lipid accessible surface area (LASA) for dimerized configurations obtained from the six SPs. Asterisks (*) denote statistical significance ($p < 0.05$) with respect to the WT. (C) Inter-monomeric van-der-Waals interaction energy for dimerized configurations of STIM1-TM WT and STIM1 M215G, M215S and I222G averaged over dimerized configurations obtained from the six SPs.

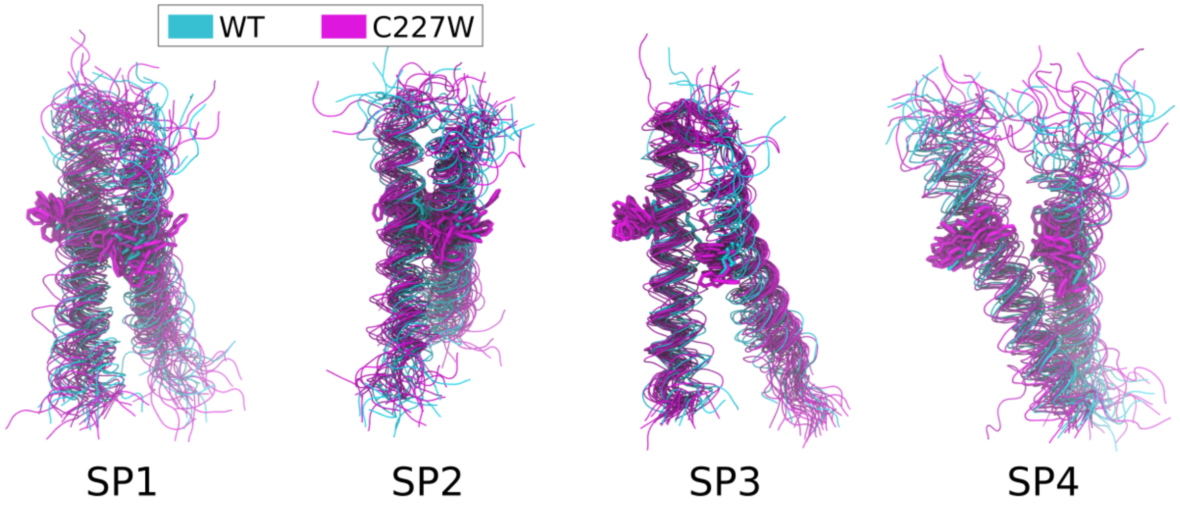

Fig. S13. Bound state conformations resulting from committor shots from 4 selected SPs for the WT (cyan) and the C227W mutant (magenta). Position 227 is highlighted in licorice representation.

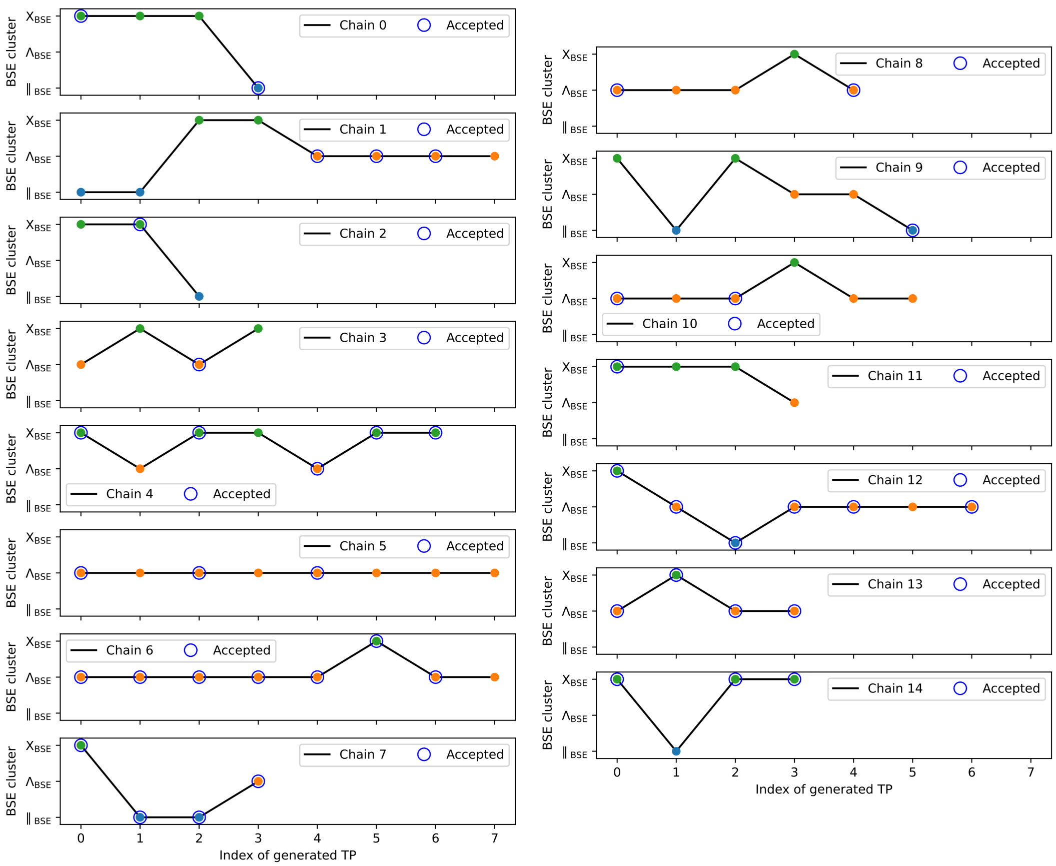

Fig. S14. Sequence of sampled bound state cluster in each of the 15 MC chains. Accepted TPs are marked by blue circles.

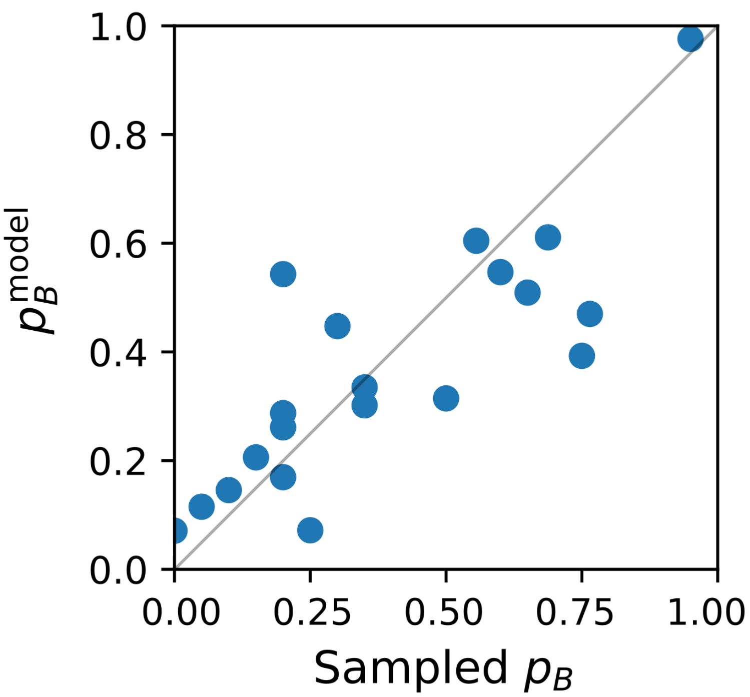

Fig. S15. Comparison of sampled p_B and p_B^{model} calculated in the distilled model, which depends only on σ(G225:G225') and σ(V217:V217').

Movie S1. Example transition path feeding into the X shaped dimerized configuration. Individual amino acids in the two STIM1-TM helices are highlighted in blue when they are within a cutoff distance of 4.5 Å of the opposing monomer. Intervening lipids are highlighted in licorice representation. Lipid phosphate groups are indicated by yellow spheres.

Movie S2. Example transition path feeding into the Λ shaped dimerized configuration. Individual amino acids in the two STIM1-TM helices are highlighted in blue when they are within a cutoff distance of 4.5 Å of the opposing monomer. Intervening lipids are highlighted in licorice representation. Lipid phosphate groups are indicated by yellow spheres.

Movie S3. Example transition path feeding into the || shaped dimerized configuration. Individual amino acids in the two STIM1-TM helices are highlighted in blue when they are within a cutoff distance of 4.5 Å of the opposing monomer. Intervening lipids are highlighted in licorice representation. Lipid phosphate groups are indicated by yellow spheres.
